# Global datasets of land surface state changes associated with the Madden-Julian Oscillation

**DOI:** 10.1016/j.dib.2019.104719

**Published:** 2019-10-24

**Authors:** Jialin Lin, Taotao Qian

**Affiliations:** Atmospheric Science Program, The Ohio State University, 154 North Oval Mall, Columbus, OH, 43210, USA

**Keywords:** Surface air temperature, Diurnal temperature range, Surface precipitation, Snow cover, Soil moisture, Madden-julian oscillation

## Abstract

This data article reports the global datasets of land surface state changes including daily maximum and minimum temperature, diurnal temperature range, surface precipitation, snow cover, soil moisture and outgoing longwave radiation associated with the Madden-Julian Oscillation (MJO), which are related to the research article entitled “Variation of Global Diurnal Temperature Range Associated with the Madden-Julian Oscillation” published in the Journal of Atmospheric and Solar-Terrestrial Physics by Lin and Qian (2019). The changes of surface air temperature and diurnal temperature range are calculated from two datasets: the Berkeley surface air temperature and the National Oceanic and Atmospheric Administration (NOAA) Climate Prediction Center (CPC) surface air temperature. The change of surface precipitation is derived from the NOAA CPC daily surface precipitation. The change of snow cover is calculated from the MODIS satellite data. The change of soil moisture is derived from the European Space Agency combined satellite data. The change of outgoing longwave radiation is calculated from NOAA satellite measurements. All of the data are stored in separate netcdf files and deposited at PANGAEA. These datasets can be used as observational benchmarks for evaluating the MJO simulations in global climate models, and in studies of MJO's impacts on global physical systems, public health, and ecosystems.

**Table:** Specifications Table

Subject	Atmospheric Science
Specific subject area	Climate Variability
Type of data	NETCDF
How data were acquired	Global land surface measurements and satellite observations
Data format	Raw Analyzed Filtered
Parameters for data collection	Data are used for December-January-February (DJF) and June-July-August (JJA).
Description of data collection	Data are collected from the Berkeley Earth Data Archive: http://berkeleyearth.org, ESA Data Archive http://www.esa-soilmoisture-cci.org, and NOAA ESRL/PSD Climate Data Archive https://www.esrl.noaa.gov/psd/.
Data source location	Globally distributed
Data accessibility	Repository name: PANGAEA Data identification number: PDI-21664 Direct URL to data: https://doi.org/10.1594/PANGAEA.906885
Related research article	Lin, J.L., and T. Qian, Variation of Global Diurnal Temperature Range Associated with the Madden-Julian Oscillation. Journal of Atmospheric and Solar-Terrestrial Physics, DOI

**Table:** 

**Value of the Data** • These datasets provide a comprehensive description of the global land surface state changes associated with the Madden-Julian Oscillation • These datasets provide the observational benchmarks for the global climate modelling community to evaluate the MJO simulations in global climate models • These datasets are also valuable for future studies on the MJO impacts on global physical systems, public health, and ecosystems

## 1 Data

The data files are deposited at PANGAEA https://doi.org/10.1594/PANGAEA.906885.

All data files are in netcdf format.

Filename convention:

Variable_Origin_cormjoSeason.nc: lag-correlation coefficient with MJO index
Variable_Origin_cormjoSeason95.nc 95% confidence level of lag-correlation coefficient

Variables:

Tmax: Daily maximum temperature
Tmin: Daily minimum temperature
DTR: Diurnal temperature range
Precip: Surface precipitation
OLR: Outgoing longwave radiation
Snow_Cover: Surface snow cover
Soil_Moisture: Soil moisture

Origin:

Berkeley, CPC, MODIS, ESA, NOAA

Season:

DJF, JJA

## 2 Experimental design, materials, and methods

The daily maximum temperature and the daily minimum temperature come from two datasets: the Berkeley surface air temperature [8] and the NOAA CPC surface air temperature [6]. The diurnal temperature range (DTR) is defined as the difference between the daily maximum temperature and the daily minimum temperature. The surface precipitation comes from CPC daily precipitation [1]. The outgoing longwave radiation (OLR) comes from NOAA daily OLR [4]. The surface snow cover is from the Moderate Resolution Imaging Spectrometer (MODIS) satellite daily snow cover [3]. The soil moisture comes from European Space Agency (ESA) Climate Change Initiative (CCI) combined satellite data, which merges measurements from SSMR, SSMI, TMI, AMSR, WindSat, SCAT, ASCAT, SMOS, and SMAP satellites [2].

The MJO index used in this calculation is the real-time multivariate MJO index principal component 1 (RMM1; [9]. Low frequency variability is first removed from all datasets using a 91-day running mean filter. The anomalies are then filtered with a 40–60 day butterworth filter [5]. Lag-correlation is calculated with the MJO index for different seasons: spring (March–May; MAM), summer (June–August; JJA), autumn (September–November; SON) and winter (December–February; DJF). Statistical significance is evaluated following [7]; which takes into account the reduced degree of freedom due to autocorrelation of the time series.
